# The immune checkpoint expression in the tumor immune microenvironment of DLBCL: Clinicopathologic features and prognosis

**DOI:** 10.3389/fonc.2022.1069378

**Published:** 2022-12-06

**Authors:** Jiajia Ma, Xuelian Pang, Junna Li, Wei Zhang, Wenli Cui

**Affiliations:** Department of Pathology, The First Affiliated Hospital, Xinjiang Medical University, Xinjiang, Urumqi, China

**Keywords:** PD-1, LAG-3, TIM-3, TIGIT, DLBCL, immune checkpoint

## Abstract

**Background & aims:**

The immune checkpoint recently provides a new strategy for the immunotherapy of malignant tumors. However, the role in the immune microenvironment of DLBCL is not completely clear.

**Methods:**

We detected the expression of PD-1, LAG-3, TIM-3, and TIGIT on TILs and on tumor cells among 174 DLBCL patients by IHC.

**Results:**

In TILs, the positive rates of PD-1, LAG-3, TIM-3 and TIGIT were 79.3%, 78.8%, 62.7% and 69.5%, respectively.TIM-3 and TIGIT were expressed in 44.8% and 45.4% of tumor cells. The expression of TIM-3 in TILs was significantly correlated with the Ann-Arbor stage (P=0.039). There was a positive correlation Between PD-1 and LAG-3 or TIM-3 and TIGIT.In addition, LAG-3 expression in TILs was associated with inferior prognosis.Multivariate analysis showed that PS score and R-CHOP therapy were independent risk factors for OS and PFS in patients with DLBCL (P=0.000).

**Conclusions:**

The expression level of TIM-3 is closely related to the Ann-Arbor stage, which may be expected to be a new index to evaluate the invasiveness of DLBCL. PD-1 was correlated with the expression of LAG-3, and the high expression of LAG-3 and LAG-3/PD-1 predicted the poor prognosis of DLBCL. Therefore, LAG-3 may become a new target of immunotherapy, or be used in combination with PD-1 inhibitors to improve the drug resistance of current patients with DLBCL.

## Introduction

In recent years, suppressive therapies targeting immune checkpoints in solid tumors have made great progress. The expression of the immune checkpoint on tumor-infiltrating lymphocytes (TILs) can exhaust CD4+ and CD8+T cells and lose their original immune killing function, thus promoting the occurrence and development of tumors.Programmed cell death 1 (PD-1) inhibitors have significantly improved patient outcomes in relapsed and refractory Hodgkin lymphoma (1, 2).The mechanisms of the PD-1/PD-L1 pathway affecting immune escape of tumor cells mainly include chromosome 9P24.1 gain and enhanced Janus Kinase(JAK)-signal transducer and activator of transcription(STAT) signal, called interferon-responsive factors (IRFs). They drive the expression of PD-L1/PD-L2, which then bind to PD-1 on the surface of anti-T cells, reducing immune cell function.In Diffuse Large B-cell lymphoma (DLBCL), R-CHOP is still the predominant treatment regimen, with a response rate of 80%, but some patients do not benefit due to clinical resistance. Even though PD-1 is widely expressed in TILs and is associated with poor survival outcomes (3). PD-1 immunosuppressive therapy does not respond well in DLBCL patients (4). Therefore, we need to seek new immune checkpoints and achieve the desired therapeutic effect through combined blockade therapy.

In the immunotherapy of malignant tumors, new potential targets include Lymphocyte-activation-gene-3(LAG-3), T cell immunoglobulin and mucin-containing molecule 3 (TIM-3), and T cell immune receptor with Ig and ITIM domains(TIGIT), their clinical significance in DLBCL is unclear.LAG-3 is a type I transmembrane protein encoded by the LAG-3 gene. It has a higher affinity with major histocompatibility complex II and is mainly expressed in activated T cells, NK cells, and Treg cells, which negatively regulates T cell proliferation and activation and mediates T cell exhaustion. Fibrinogen like protein 1(FGL-1) is an important ligand of LAG-3 and induces a significantly reduced antitumor response (5).Therefore, high expression of LAG-3(LAG-3high) is often associated with poor survival (6). LAG-3 is often expressed in conjunction with other immune checkpoints and plays a role in the immune escape, the most common of which is PD-1. In addition, LAG-3 expression in TILs was also demonstrated in follicular lymphoma (FL) and Hodgkin’s lymphoma(HL) (6, 7) and also showed good response to dual blockade PD-1/LAG-3 treatment (8).

TIM-3 belongs to the TIM gene family. The TIM family includes TIM-1, TIM-3, and TIM-4, located on chromosome 5q33.2.TIM-3 inhibits tumor immunity by stopping the helper T(Th) cell immune response primarily by recognizing the ligand Galectin- 9 (9).TIM-3 expression can be detected in some solid tumors and can be used as a therapeutic target or prognostic indicator (10).In hematological tumors, TIM-3 overexpression was found in Regulatory T cell (Treg) in peripheral blood of Chronic Lymphocytic Leukemia(CLL) patients, which promoted the binding with galectin-resulting in Treg/Th17 imbalance and decreased immune function (11). TIM-3 is not only expressed in TILs, but also detected at certain levels in tumor cells, but the exact mechanism is still indefinite (12).

TIGIT is a co-inhibitory receptor in the Ig superfamily and expresses by activated T cells, Treg, and NK cells. Negative regulation of T cells can be achieved by blocking the activation of costimulatory receptors and affecting the function of Treg cells. Under normal circumstances, the expression of TIGIT is considered to properly regulate the autoimmune response. However, under long-term antigen (such as malignant tumor) stimulation, the continuous expression of TIGIT leads to the exhaustion of T cell function, resulting in the deficiency of immune function. TIGIT overexpression has been detected in T cells of CLL, Sezary syndrome, and Acute Myelocytic Leukemia(AML) (13–15). TIGIT is often co-expressed with PD-1 and other immune checkpoints on CD8+TILs.Dual PD-1/TIGIT blockers have achieved higher clinical efficacy in melanoma compared with single immune checkpoint inhibition confirming the potential possibility of TIGIT as an immunotherapy target (16, 17).

## Materials and methods

### Study population and materials

This retrospective study enrolled the clinicopathological information of 174 DLBCL patients at the Department of Pathology, First Affiliated Hospital of Xinjiang Medical University between 2012 and 2017. All patients were diagnosed and classified by two senior Pathologists according to the 2016 version of the World Health Organization Pathology and Genetics of Hematopoietic and Lymphoid Tissue Neoplasms (18). The clinicopathological characteristics of patients, including age, sex, tumor site, subtype, Ann-Arbor stage, IPI, performance status (PS), symptom B (unexplained fever >38°C, night sweats, and weight loss >10% within 6 months), bone marrow involvement, serum lactate dehydrogenase (LDH) level, treatment and follow-up results, etc. The longest follow-up period was 96 months. This study was approved by the Medical Ethics Committee of the First Affiliated Hospital of Xinjiang Medical University.

Corresponding sections and formalin fixation and paraffin embedding (FFPE) tissue of included patients were collected from the medical record database of the Department of Pathology, The First Affiliated Hospital of Xinjiang Medical University. All HE sections were selected from two representative areas by two senior pathologists and labeled at the same location of the corresponding wax block. The labeled tissues were perforated from the wax block with sampling needles and vertically implanted into the prepared recipient wax block. The tissue were sequentially sliced with a thickness of 4 microns and attached to the slides for subsequent immunohistochemical experiments.

### Immunohistochemistry staining

**Figure 1:**
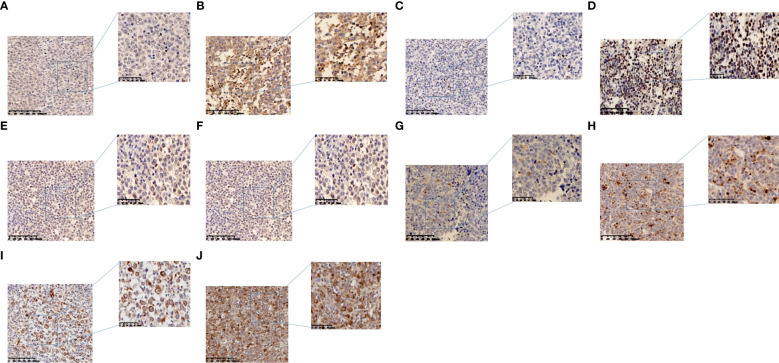
Representative images of immunohistochemistry in DLBCL. **(A)** Low PD-1 expression in TILs. **(B) **High PD-1 expression in TILs. **(C)** Low LAG-3 expression in TILs. **(D) **High LAG-3 expression in TILs. **(E)** Low TIM-3 expression in TILs. **(F)** High TIM-3 expression in TILs. **(G)** Low TIGIT expression in TILs. **(H)** High TIGIT expression in TILs. **(I)** TIM-3 expression in tumor cells. **(J)** TIGIT expression in tumor cells.

Immunohistochemistry staining was used to detect the expression of PD-1, LAG-3, TIM-3, and TIGIT proteins in tissue microarray. First, the slides were placed on a baking apparatus at 70°C for 1h, then dewaxed by xylene (for 10 min, twice), dehydrated by ethanol gradient, and then cleaned with distilled water for 5min. Subsequently, the slides were incubated with H2O2 for 10min at room temperature to eliminate endogenous peroxidase activity. EDTA repair buffer (pH 8.0) was repaired in boiling water for 20min. Cool to room temperature and incubate in goat serum (Beijing Zhongshan Golden Bridge Biological Technology) and incubated at 37˚C for 20 min. Primary anti-PD-1 (18106-1-AP,Proteintech,1:1000),LAG-3(16616-1-AP,Proteintech,1:1200),TIM-3 (clone 4C4G3,60355-1-Ig,Proteintech,1:800),TIGIT(clone BLR047F,ab243903,Abcam,1:300) were added,overnight at 4˚C. The tissue chips were washed with PBS for 5min and incubated with a secondary antibody that was added dropwise (PK 10006 universal kit, Proteintech) for 30min at 37˚C. The sections were stained for the 40s with hematoxylin (Beijing Zhongshan Golden Bridge Biological Technology), terminated with tap water, differentiated with hydrochloric acid ethanol for 5s, blue back with PBS for 5min, and washed with tap water for 1min. Gradient ethanol for rapid dehydration, using xylene transparent, neutral resin closed slides. IHC staining was scored blindly by two independent pathologists according to the proportion of PD-1, LAG-3, TIM-3, TIGIT positive cells, and staining intensity. Tumor infiltrating lymphocytes (TILs) or tumor cells that were positive for immune checkpoint were enumerated by recording the average number of positive lymphocytes in 2-3 regions with the highest 40-fold density. Staining intensity was scored as follows: i) 0, negative; ii) 1, weak; iii) 2, moderate; iv) 3, intense. The extent of staining was scored as follows: i) 0, 0% of tumor area stained; ii) 1, <20%; iii) 2, 20-50%; iv) 3, >50%. The case with the final score of 0 is regarded as negative, and the other scores are regarded as positive. In addition, scores of 0-3 and 4-9 are defined as low expression and high expression, respectively. The scoring system is similar to previous studies (19, 20). (**Figure 1**).

### Statistical analysis

The statistical analysis was performed using SPSS 22.0. The clinical relevance of PD-1, LAG-3, TIM-3, TIGIT, and other clinicopathologic data were evaluated using the Chi-square test and rank sum test. Spearman correlation between the two factors analysis.Kaplan–Meier curves with the log-rank test were employed to analyze the survival data. The Cox regression model was applied to multivariate analysis. P ≤ 0.05 was considered significant.

## Results

### Clinicopathological characteristics of DLBCL patients

A total of 174 DLBCL patients were included in this study, including 95 males (54.6%) and 79 females (45.4%)(male: female ratio,1.2:1). Patients ranged in age from 5 to 87 years, with a median age of 59 years.25.3% patients had type B symptoms. The LDH was normal (109-250IU/L) in 83 cases (47.7%), higher than normal (>250IU/L) in 56 cases (32.2%). According to Ann-Arbor staging criteria, 39 cases (22.4%) were stage I and II, and 112 (64.4%) were stage III and IV.The international prognostic index (IPI) score was 0-1 in 55 cases (31.6%), 2-3 in 96 cases (55.2%),4-5 in 9 cases (5.2%), and bone marrow involvement in 24 cases (13.8%).There were 128 patients (73.6%) with tumor diameter ≤5cm, and 40 patients (23.0%) with tumor diameter > 5cm.107 (61.5%) and 60 (34.5%) patients had extra-and intra-nodal lymphoma, respectively.42 patients (24.1%) were diagnosed with germinal center B-cell-like lymphoma(GCB) lymphoma and 127 patients (73.0%) were diagnosed with Non-GCB lymphoma (**Table 1**). 71 patients survived, 45 died, and 58 were lost to follow-up. The 174 patients had a median overall survival of 32 months and a median progression-free survival of 31 months.

**Table 1:** Clinical relevance of PD-1, LAG-3 , TIM-3 and TIGIT expressions on TILs.

Characteristics		Low			High		P	Low			High		P	Low		High		P		Low		High		P
Gender																								
Male		62			33		0.739	52			40		0.284	64		24		0.748		49		36		0.381
Female		50			29			51			28			50		21				37		36		
Age, years																								
≤60		54			35		0.298	50			38		0.347	60		24		0.936		45		36		0.771
>60		58			27			53			30			54		21				41		36		
Ann-Arbor stage																								
I and II		29			10		0.151	21			17		0.247	32		5		0.039*		23		14		0.362
III and IV		69			43			73			38			69		31				54		47		
IPI																								
Low risk (0-1 score)		37			18		0.893	35			20		0.941	42		13		0.643		29		26		0.904
Medium risk (2-3 score)		61			35			58			36			58		26				48		37		
High risk (4-5 score)		6			3			6			3			5		2				4		3		
Performance State																								
≤2 score		98			52		0.732	93			55		0.418	98		39		0.686		77		62		0.928
PD-1 expression																								
LAG-3 expression																								
TIM-3 expression																								
TIGIT expression																								
>2 score		6			4			5			5			7		2				4		3		
LDH, u/l																								
≤250		52			31		0.986	54			27		0.080*	55		20		0.566		45		31		0.094
>250		35			21			29			27			35		16				22		28		
B symptom																								
Yes		26			18		0.166	25			17		0.757	23		15		0.101		24		16		0.494
No		75			31			66			40			74		25				42		45		
Location																								
Extranodular		71			36		0.411	69			36		0.062	66		29		0.303		49		46		0.333
Intranodular		36			24			30			29			44		13				34		23		
Diameter, cm																								
≤5		82			46		0.914	77			48		0.458	79		34		0.359		59		53		0.599
>5		26			14			22			18			31		9				23		17		
Subtypes																								
GCB		29			13		0.535	28			14		0.343	33		8		0.150		24		16		0.505
Non-GCB		81			46			73			52			79		36				62		53		
Extranodal sites																								
≤2		84			43		0.673	72			52		0.178	83		32		0.323		65		49		0.374
>2		25			15			28			12			26		11				18		19		
Bone Mallow Needle Biopsy																								
Yes		9			15		0.006*	11			13		0.050*	14		5		0.762		13		7		0.178
No		81			39			79			39			78		33				54		57		
R-CHOP treatment																								
Yes		47			26		0.898	43			30		0.227	51		18		0.534		36		33		0.228
No		51			27			52			24			47		21				43		26		

IPI, International Prognostic Index; PS, performance status; LDH,lactate dehydrogenase; *P≤0.05 was statistically significant.

### The expression of PD-1,LAG-3,TIM-3,TIGIT on DLBCL tissues

PD-1, LAG-3, TIM-3, TIGIT are widely expressed in the lymphocytes of TILs. This study did not use multiple staining protocol because large lymphoma cells and lymphocytes have its own morphology characteristics so they can be easily distinguished.PD-1 showed positive staining on TILs in 79.3% of DLBCL cases and LAG-3 showed positive staining on TILs in 78.8% of DLBCL cases.Both TIM-3 and TIGIT were positive on TILs in DLBCL cases (62.7% and 69.5%, respectively), but were also expressed on tumor cells(44.8% and 45.4%, respectively). Only a small amount of PD-1 and LAG-3 were expressed in tumor cells, with positive rates of 4.0% and 2.9%, respectively(**Figure 1**, **Table 2**).

**Table 2:** IHC analysis of DLBCL cases.

Immunohistochemical Staining	In TILs				In Tumor cells			
	The expression in TILs		Scoring situation		The expression in Tumor cells		Scoring situation	
(N=174)	positive(>0 score)	negative(0 score)	Low expression(0-3 score)	High expression(4-9 score)	positive(>0 score)	negative(0 score)	Low expression(0-3 score)	High expression(4-9 score)
PD-1	(138) 79.3%	(36) 20.7%	(112)64.4%	(62) 35.6%	(7) 4.0%	(167) 96.0%		
LAG-3	(137)78.8%	(37) 21.2%	(108) 62.1%	(66)37.9%	(5)2.9%	(169 )97.1%		
TIM-3	(109) 62.7%	(65)37.3%	(135)77.6%	(39) 22.4%	(78 )44.8%	(96) 55.2%	(157) 90.2%	(17) 9.8%
TIGIT	(121)69.5%	(53) 30.5%	(107 )61.5%	(67)38.5%	(79)45.4%	(95) 54.6%	(161) 92.5%	(13) 7.5%
								

Scoring situation of PD-1 and LAG-3 are not included in the table due to too few positive expression cases in tumor cells.

### The association between PD-1, LAG-3, TIM-3, TIGIT expression and clinicopathology in DLBCL

The expression of PD-1 and LAG-3 in TILs was significantly correlated with bone marrow involvement (P=0.006, P=0.050), and the expression of LAG-3 in TILs was also correlated with LDH content (P=0.080).The expression of TIM-3 in TILs was significantly correlated with Ann-Arbor stage (P=0.039). The expression of TIM-3 on tumor cells was significantly correlated with the occurrence of symptom B (P=0.019).There was no correlation between other immune checkpoints and pathological features (P>0.05) (**Tables 1**, **3**).

**Table 3:** Clinical relevance of TIM-3 and TIGIT expressions on tumor cells.

	TIM-3 tumor			TIGIT tumor			
Clinicopathological characteristics	Low	High	P	Low		High	P
Gender							
Male	65	19	0.547	73		12	0.201
Female	49	18		57		16	
Age, years							
≤59	61	20	0.954	68		13	0.572
>59	53	17		62		15	
Ann-Arbor stage							
I and II	27	9	0.952	33		4	0.177
III and IV	74	24		80		21	
IPI							
Low risk (0-1 score)	35	18	0.213	43		12	0.482
Medium risk (2-3 score)	66	17		72		13	
High risk (4-5 score)	5	2		5		2	
Performance state							
≤2 score	98	36	0.296	112		27	0.196
>2 score	8	1		7		0	
LDH, u/l							
≤250	56	15	0.908	68		8	0.230
>250	39	11		41		9	
B symptom							
Yes	22	15	0.019*	32		8	0.956
No	77	20		78		19	
Location							
Extranodular	69	20	0.682	80		15	0.578
Intranodular	41	14		46		11	
Diameter, cm							
≤5	81	27	0.935	91		21	0.861
>5	28	9		33		7	
Subtypes							
GCB	30	10	0.907	30		10	0.186
Non-GCB	82	26		97		18	
Extranodal sites							
≤2	82	28	0.696	92		22	0.425
>2	28	8		32		5	
Bone Mallow Needle Biopsy							
Yes	16	2	0.166	18		2	0.230
No	79	28		87		24	
R-CHOP treatment							
Yes	48	19	0.340	57		12	0.825
No	52	14		56		13	

PD-1 TILs and LAG-3 TILs were correlated (r=0.204, P=0.008), TIM-3 TILs and TIGIT TILs were correlated (r=0.158,P=0.050), and TIM-3 tumor and TIGIT tumor were correlated (r=0.198, P=0.017).However, no correlation was found between the expression of the same immune checkpoint in TILs and tumor cells (**Table 4**).

**Table 4:** Spearman correlation analysis of PD-1, LAG-3, TIM-3, TIGIT expression.

LAG-3 expression				
PD-1 expression	Low (%)	High (%)	R	P-value
Low (%)	75	36	0.204	0.008*
High (%)	28	32		
TIM-3 expression				
TIGIT expression	Low (%)	High (%)	R	P-value
Low (%)	67	17	0.158	0.050*
High (%)	46	24		
TIM-3 tumor expression				
TIGIT tumor expression	Low (%)	High (%)	R	P-value
Low (%)	94	16	0.198	0.017*
High (%)	25	12		

*P ≤ 0.05 was statistically significant.

### Survival analysis of pathological features and immune checkpoint expression

**Figure 2:**
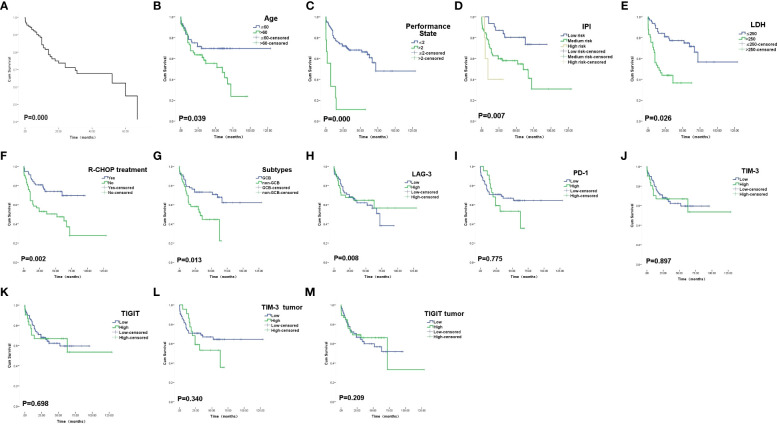
Overall survival curves. **(A)** The overall COX survival curves of these 174 patients with DLBCL. **(B)** Survival curves of patients according to Age. **(C)** Survival curves of patients according to PS score. **(D)** Survival curves of patients according to IPI index. **(E)** Survival curves of patients according to LDH. **(F)** Survival curves of patients according to R-CHOP treatment. **(G)** Survival curves of patients according to subtypes. **(H)** Survival curves of patients according to LAG-3 expression on TILs. **(I)** Survival curves of patients according to PD-1 expression on TILs. **(J)** Survival curves of patients according to TIM-3 expression onTILs. **(K)** Survival curves of patients according to TIGIT expression on TILs. **(L)** Survival curves of patients according to TIM-3 expression on tumor cells. **(M)** Survival curves of patients according to TIGIT expression on tumor cells.

**Figure 3:**
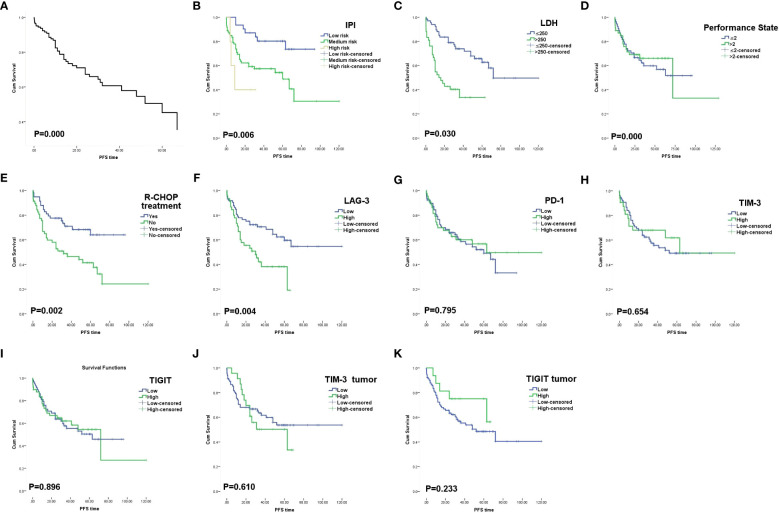
Progression free survival curves. **(A)**The progression free survival COX survival curves of these 174 patients with DLBCL. **(B)** Survival curves of patients according to IPI index. **(C)** Survival curves of patients according to LDH. **(D)** Survival curves of patients according to PS score. **(E)** Survival curves of patients according to R-CHOP treatment. **(F) **Survival curves of patients according to LAG-3 expression on TILs.**(G)** Survival curves of patients according to PD-1 expression on TILs. **(H)** Survival curves of patients according to TIM-3 expression onTILs. **(I)** Survival curves of patients according to TIGIT expression on TILs. **(J)** Survival curves of patients according to TIM-3 expression on tumor cells. **(K)** Survival curves of patients according to TIGIT expression on tumor cells.

Progression-free survival (PFS) was defined as the time from randomization to tumor progression (any aspect) or death (from any cause). Overall Survival (OS) was defined as the time from randomization to death from any cause (last follow-up for patients lost to follow-up; end-of-follow-up day for patients still alive at the end of the study). Among the pathological features,Age>60 (P= 0.039), higher IPI (P=0.007), Performance State >2 points (P=0.000), LDH> 250U/L (P=0.026) showed shorter OS, while R-CHOP treatment was associated with longer OS(P=0.002).Non-GCB showed a worse prognosis than GCB (P=0.013) (**Figure 2**). Higher IPI (P=0.006), Performance State >2 points (P=0.000), LDH> 250U/L (P=0.030) showed shorter PFS, while R-CHOP treatment was associated with longer PFS (P=0.002) (**Figure 3**).

**Figure 4:**
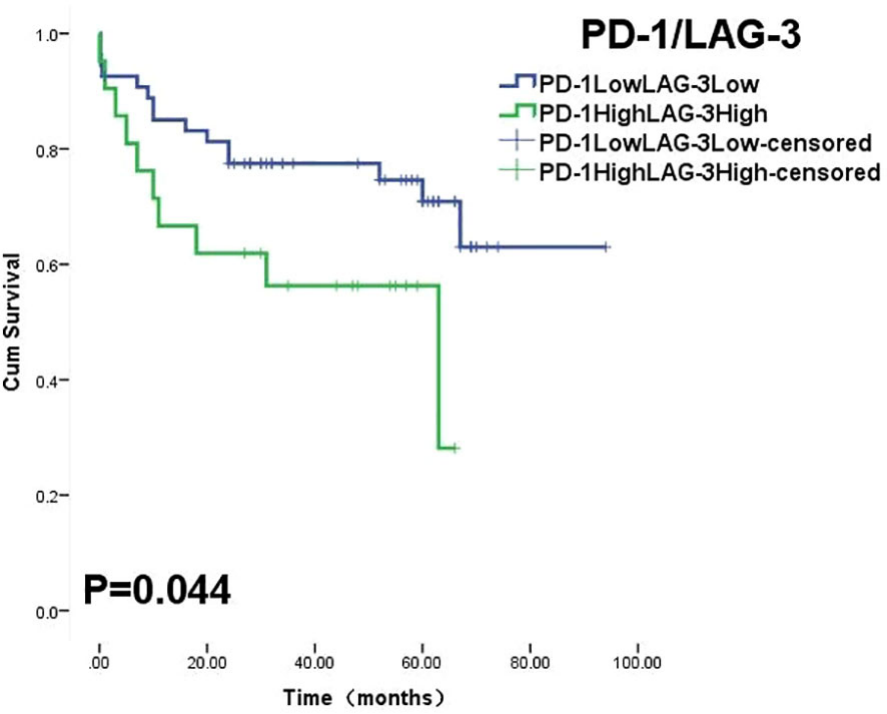
Overall survival curves was stratified by PD-1 and LAG-3 co-expression on TILs.

The 5-year OS and PFS of high and low expression of LAG-3 TILs were 46.5% vs 69.1%(OS, P=0.011) and 40.0% vs 63.0%(PFS, P=0.006), respectively, with statistically significant differences. In TILs, LAG-3LowPD-1Low had a longer survival time than LAG-3High PD-1High (P=0.044) (**Figure 4**). There was no significant difference in the expression and prognosis of PD-1 TILs, TIM-3 TILs, and TIGIT TILs (P>0.05).In addition, the expression of TIM-3 and TIGIT in tumor cells and prognosis were not statistically significant(P>0.05). In multivariate analysis, Performance State score and R-CHOP treatment were independent risk factors affecting OS and PFS in DLBCL patients(P=0.000) (**Tables 5**, **6**).

**Table 5:** Univariate and multivariate analyses of clinicopathological factors associated with overall survival in DLBCL patients.

	Univariate analysis				Multivariate analysis			
Clinicopathological characteristics	HR	95% CI		p-value	HR	95% CI		p-value
Gender	O.973	0.540	1.752	0.926				
Age, years	1.853	1.020	3.369	0.039	1.556	0.818	2.959	0.177
Ann-Arbor stage	1.358	0.650	2.837	0.416				
IPI	2.920	1.347	6.330	0.007	1.915	0.764	4.801	0.166
Performance state	6.028	2.735	13.287	0.000	5.458	2.082	14.307	0.001*
LDH, u/l	3.769	1.957	7.256	0.026	3.472	1.723	6.998	0.122
B symptom	0.678	0.355	1.295	0.239				
Location	0.922	0.504	1.687	0.792				
Diameter, cm	0.966	0.464	2.013	0.927				
Subtypes	2.833	1.192	6.738	0.013	2.099	0.860	5.123	0.104
Extranodal sites	0.647	0.551	2.606	1.199				
Bone marrow involvement	1.145	0.447	2.931	0.778				
R-CHOP treatment	2.577	1.385	4.795	0.002	2.181	1.086	4.379	0.028*
PD-1	0.914	0.491	1.701	0.775				
LAG-3	2.212	1.204	4.065	0.008	0.581	0.295	1.144	0.116
TIM-3	1.048	0.517	2.121	0.897				
TIGIT	0.882	0.465	1.670	0.699				
TIM-3 tumor	1.409	0.692	2.869	0.345				
TIGIT tumor	0.522	0.185	1.472	0.219				

*P ≤ 0.05 was statistically significant.

**Table 6:** Univariate and multivariate analyses of clinicopathological factors associated with progression free survival in DLBCL patients.

	Univariate analysis				Multivariate analysis			
Clinicopathological characteristics	HR	95% CI		p-value	HR	95% CI		p-value
Gender	0.952	0.559	1.620	0.856				
Age, years	1.550	0.911	2.638	0.106				
Ann-Arbor stage	1.315	0.675	2.563	0.421				
IPI	1.987	1.090	3.622	0.006	1.513	0.746	3.070	0.251
Performance State	5.277	2.441	11.407	0.000	3.913	1.579	9.698	0.003*
LDH, u/l	3.433	1.895	6.218	0.030	3.057	1.601	5.838	0.154
B symptom	0.801	0.446	1.438	0.458				
Location	1.109	0.644	1.910	0.710				
Diameter, cm	1.067	0.561	2.030	0.843				
Subtypes	1.325	0.690	2.545	0.060				
Extranodal sites	1.896	0.974	3.690	0.398				
Bone marrow involvement	1.198	0.509	2.820	0.679				
R-CHOP treatment	2.350	1.330	4.153	0.002	2.609	1.412	4.819	0.024*
PD-1	0.929	0.532	1.621	0.795				
LAG-3	2.160	1.250	3.732	0.004	0.531	0.283	0.995	0.048
TIM-3	0.862	0.447	1.661	0.862				
TIGIT	0.963	0.547	1.697	0.963				
TIM-3 tumor	1.186	0.613	2.295	0.613				
TIGIT tumor	0.575	0.228	1.452	0.242				

*P≤0.05 was statistically significant.

## Discussion

DLBCL is the most common type of Non-Hodgkin’s lymphoma(NHL) and is highly invasive and heterogeneous in clinical presentation and prognosis.The therapeutic effect of PD-1/PD-L1 inhibitors in DLBCL is not significant.Compared with reactive hyperplasia lymph nodes, PD1+CD8+T cells were often increased in DLBCL.These CD8+T cells are almost universally activated and functional in DLBCL, even in the presence of the expression of negative regulatory molecules such as PD-1 and TIM-3, but lack the main feature of depletion, which PD-1 inhibitors require in order to function (21).In addition, it has also been observed that PD-1 shows high expression on Treg, and anti-PD-1 antibodies may lead to increased activity of Treg, leading to suppression of immune response (22).These findings may explain the current failure of immune checkpoint therapy.Therefore, we urgently need to find new immune checkpoint inhibitors to improve the treatment status of DLBCL.

After detecting the expression of PD-1 and LAG-3 in DLBCL tissues in 174 patients,we found that PD-1 and LAG-3 were widely expressed in TILs (**Figure 1**) and were correlated with bone marrow involvement.The expression of LAG-3 was also different at high and low LDH levels (**Table 1**). Studies have indicated that the expression level of LAG-3 in relapsed DLBCL patients is up-regulated compared with that of newly diagnosed DLBCL patients, and it is related to PS score (23), suggesting that the high expression of PD-1 and LAG-3 immune checkpoints may be related to the aggressiveness of DLBCL.In addition, we found a correlation between the expression level of PD-1 and LAG-3 protein (**Table 4**), indicating that there are some cascades between PD-1 and LAG-3 in the co-inhibitory receptor pathway, which jointly maintain immune microenvironment homeostasis.At the same time, under the long-term stimulation of tumor cells, PD-1+LAG-3+T cells further exhaust, and their ability to produce cytokines and cytotoxic particles is greatly reduced (24). Most importantly, we found that high expression of LAG-3 in TILs was associated with lower survival in DLBCL patients (**Figures 2**, **3**). The 5-year PFS and OS were also longer for LAG-3Low PD-1Low than for LAG-3High PD-1High (**Figure 4**). This was similar to the results of Keane, who showed that DLBCL patients had high LAG-3 expression in TILs, along with enrichment of PD-1 and TIM-3, and that high LAG-3 gene expression was associated with low survival in the cohort (25). For HL, combined blocking of LAG-3 and PD-1 has stronger anti-tumor immunity mediated by CD4+T cells than single treatment (8). Although there was no significant correlation between PD-1 and prognosis in this study, which may be related to different experimental standards or insufficient sample size, LAG-3 or PD-1/LAG-3 combination is still promising as a new immunosuppressive target.

Unlike the first two immune checkpoints, TIM-3 was expressed in tumor cells at a rate of 44.8% and was associated with the occurrence of symptom B (**Figure 1**, **Table 3**). We speculated that the expression of TIM-3 in tumor cells also affected the tumor microenvironment of DLBCL, which was manifested as the aggravation of clinical symptoms.In addition, TIM-3 in TILs is associated with Ann-Arbor stage (**Table 1**), which is derived from a patient’s actual tumor invasion and is commonly used to assess the malignancy of the disease.Benjamin et al. examined the expression of TIM-3 protein on tumor cells and TILs in 123 patients with DLBCL.39% of DLBCL patients showed positive TIM-3 in tumor cells, and patients with high expression of TIM-3 in TILs had a poor prognosis (12). Liu’s research shows that patients with PD-1 and TIM-3 co-expression have poor PFS stage (26). A multiplex immunofluorescence analysis showed that patients with exhausted TIM-3+CD8+T cells in DLBCL showed poor survival rate (27). The high abundance of TIM-3+Foxp3+Treg (TFT) cells in TME indicates the poor prognosis of DLBCL, and TFT cells promote the development of DLBCL by secreting IL-10 in TME (28). TIM-3 can also activate NF- κ B phosphorylation, which in turn stimulate IL-6 secretion and STAT3 phosphorylation, resulting in a decrease in the ability of immune cells to monitor and kill tumors (29). All the above results show that TIM-3 plays a negative regulatory role in DLBCL.Therefore, we think that TIM-3 may be a new index to evaluate malignant degree of DLBCL patients.

TIGIT and TIM-3 seem to have similar biological characteristics, the expression of TIGIT in tumor cells is also high, accounting for 45.4%, and the expression of TIGIT and TIM-3 proteins in TILs and tumor cells display correlated(**Figure 1**, **Table 4**). Single cell RNA sequencing analysis showed that the co-suppressed signal mediated by TIM-3 or TIGIT in DLBCL seemed to be the main driving force of T cell failure.These data suggest that TIGIT, as an inhibitory receptor, may affect the tumor microenvironment of DLBCL and negatively regulate the function of T cells together with TIM3.In NHL, it has been confirmed that TIGIT is widely expressed in T cells, and most of them have high co-expression of PD-1 to stimulate the release of inhibitory cytokines (30). We haven’t found the clue that TIGIT can be used as a new immunotherapy target yet, and further research is needed to explore its biological significance.

In summary, the expression level of TIM-3 is closely related to Ann-Arbor stage, which may be expected to be a new indicator to evaluate the aggressiveness of DLBCL.PD-1 was correlated with the expression of LAG-3, and both the high expression of LAG-3 and LAG-3/PD-1 predicted the poor prognosis of DLBCL.Therefore, LAG-3 may be a new target for immunotherapy, and it can also be combined with PD-1 inhibitors to improve the drug resistance of current DLBCL patients, thus extending the survival of patients.

## Data availability statement

The raw data supporting the conclusions of this article will be made available by the authors, without undue reservation.

## Ethics statement

Written informed consent was obtained from the individual(s), and minor(s)’ legal guardian/next of kin, for the publication of any potentially identifiable images or data included in this article.

## Author contributions

WC and WZ contributed to the study conception and design. Material preparation, data collection and analysis were performed by JM, JL and XP. The frst draft of the manuscript was written by JM and all authors commented on previous versions of the manuscript. All authors contributed to the article and approved the submitted version.
